# Visual and Olfactory Floral Cues of *Campanula* (Campanulaceae) and Their Significance for Host Recognition by an Oligolectic Bee Pollinator

**DOI:** 10.1371/journal.pone.0128577

**Published:** 2015-06-10

**Authors:** Paulo Milet-Pinheiro, Manfred Ayasse, Stefan Dötterl

**Affiliations:** 1 Institute of Experimental Ecology, University of Ulm, Ulm, Germany; 2 Department of Plant Systematics, University of Bayreuth, Bayreuth, Germany; Monash University, AUSTRALIA

## Abstract

Oligolectic bees collect pollen from a few plants within a genus or family to rear their offspring, and are known to rely on visual and olfactory floral cues to recognize host plants. However, studies investigating whether oligolectic bees recognize distinct host plants by using shared floral cues are scarce. In the present study, we investigated in a comparative approach the visual and olfactory floral cues of six *Campanula* species, of which only *Campanula lactiflora* has never been reported as a pollen source of the oligolectic bee *Ch*. *rapunculi*. We hypothesized that the flowers of *Campanula* species visited by *Ch*. *rapunculi* share visual (i.e. color) and/or olfactory cues (scents) that give them a host-specific signature. To test this hypothesis, floral color and scent were studied by spectrophotometric and chemical analyses, respectively. Additionally, we performed bioassays within a flight cage to test the innate color preference of *Ch*. *rapunculi*. Our results show that *Campanula* flowers reflect the light predominantly in the UV-blue/blue bee-color space and that *Ch*. *rapunculi* displays a strong innate preference for these two colors. Furthermore, we recorded spiroacetals in the floral scent of all *Campanula* species, but *Ca*. *lactiflora*. Spiroacetals, rarely found as floral scent constituents but quite common among *Campanula* species, were recently shown to play a key function for host-flower recognition by *Ch*. *rapunculi*. We conclude that *Campanula* species share some visual and olfactory floral cues, and that neurological adaptations (i.e. vision and olfaction) of *Ch*. *rapunculi* innately drive their foraging flights toward host flowers. The significance of our findings for the evolution of pollen diet breadth in bees is discussed.

## Introduction

When navigating the landscape, pollinators are frequently confronted with a staggering diversity of flowers in the complex floral market and have to make choices. Making the right decision (e.g. choosing resource-rich flowers) might benefit pollinators by optimizing foraging efficiency [1]. The decision may reflect either innate [2, 3] or learned preferences [4, 5] for some floral cues, which are possibly related to neurophysiological adaptations of pollinators (e.g. olfactory receptors) and their diet breadth [6–8]. For example, specialized (oligolectic) bees collect pollen only on plants of a given genus or family to rear their offspring [9, 10] and, consequently, must rely on host-specific floral cues to recognize host flowers unambiguously. Generalized (polylectic) bees, on the other hand, collect pollen on a vast array of plant species irrespective of phylogenetic relatedness, and might rely on more generalized floral cues [11].

Bees are known to use mainly visual (e.g. color) and olfactory floral cues (e.g. floral scents) to find and select food sources [12]. Generally, visual and olfactory cues work together in modulating bees' behavior, however, the relative importance each cue play varies greatly across associations (for a review see [13]). Visual cues alone are known to attract bees and are often used by them to discriminate between rewarding and non-rewarding flowers within a species [14–17]. However, as compared to floral scent, visual cues are assumed to play a minor role in the discrimination of flowers among species [4, 18]. For example, when considering color, the most well investigated visual cue, flowers of species belonging to different genera or families are often very similar and may not be well discriminated by bees [19–22]. Conversely, the potential infinite diversity of floral scents, due to either highly specific compounds or unique ratios of common compounds [23, 24], makes them highly specific cues that provide pollinators with an astonishing amount of information. Not surprisingly, there are increasing evidences that bees use floral volatiles to discriminate among flowers, both intra-specifically, such as among flowers of different plants, flowers within an inflorescence, and flowers with different amount of reward (e.g. pollen and nectar), and inter-specifically [19, 21, 25–29]. More specifically, in associations involving specialists, such as those between some oligolectic bees and their host plants, floral scents are known to be the ultimate cue for host recognition [11].

**Fig 1:**
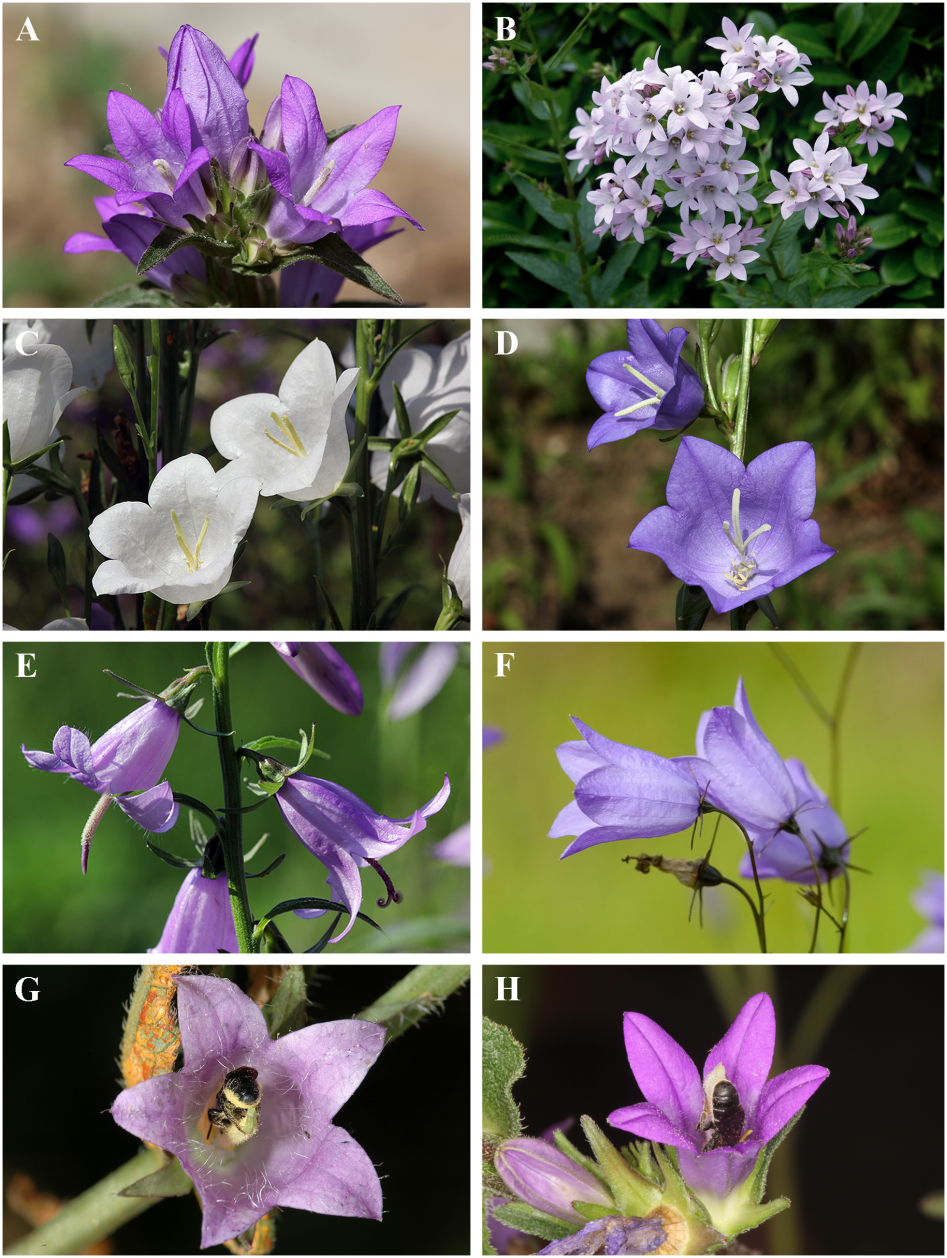
Flowers of *Campanula* species used for the study and their oligolectic pollinator *Chelostoma rapunculi*. (A) *Ca*. *glomerata*, (B) *Ca*. *lactiflora* (C) *Ca*. *persicifolia alba*, (D) *Ca*. *persicifolia*, (E) *Ca*. *rapunculoides*, (F) *Ca*. *rotundifolia*, and (G) *Ca*. *trachelium*. (G, H) Females of *Ch*. *rapunculi* gathering pollen on *Ca*. *trachelium* and *Ca*. *glomerata*, respectively. All photos by Paulo Milet-Pinheiro.

*Chelostoma rapunculi* (Lepeletier 1841) (Fig 1) is a European oligolectic bee that collects pollen on several *Campanula* species [30, 31], and in the presence of host plants, both males and females restrict nectar gathering to these plants. The genus *Campanula* L. (Campanulaceae) comprises about 400 species of bellflowers, harebells, and starbells, whose flowers are predominantly violet-blue to the human eyes [32, 33]. In spite of its remarkable diversity, floral scent composition in the genus *Campanula* has been chemically characterized only in a single species, namely *Ca*. *trachelium* [34]. Recently, we showed that *Ch*. *rapunculi* innately prefers both visual and olfactory floral cues of *Ca*. *trachelium* over those of two other co-flowering non-host plants [20]. The exact cues involved in the innate visual preference by *Ch*. *rapunculi* remain unknown, even if some evidence point to color as important visual dimension [35]. In terms of olfactory cues, however, newly-emerged bees of *Ch*. *rapunculi* were shown to rely on spiroacetals to discriminate host from non-host flowers [34]. Spiroacetals form a distinct group of natural volatiles that are rarely encountered in floral scents [24]. In the floral scent bouquet of *Ca*. *trachelium*, however, six spiroacetals were found, some of them recorded for the first time as floral scent constituents [34].

In the present study, we investigated in a comparative approach the visual (i.e. color reflectance spectra) and olfactory (i.e. scent bouquet) floral cues of five *Campanula* species which are used as pollen source by *Ch*. *rapunculi* (*Ca*. *glomerata*, *Ca*. *persicifolia*, *Ca*. *rapunculoides*, *Ca*. *rotundifolia*, and *Ca*. *trachelium*) and one species (*Ca*. *lactiflora*), which is not visited by this bee species [30, 36]. We predict that *Campanula* flowers visited by *Ch*. *rapunculi* share visual and/or olfactory cues that give them a host-specific signature, allowing bees to discriminate them from non-host flowers. More specifically, we hypothesize that 1) *Campanula* species, which are visited by *Ch*. *rapunculi*, have similar floral color reflectance, 2) *Ch*. *rapunculi* bees have an innate preference for the color of host species, and 3) Spiroacetals are constituents of the floral scent bouquet of the *Campanula* species, which are used by *Ch*. *rapunculi* as pollen source.

## Materials and Methods

### Plant species

Plant individuals of *Ca*. *lactiflora*, *Ca*. *glomerata*, *Ca*. *persicifolia* (blue and white variety), *Ca*. *rapunculoides*, *Ca*. *rotundifolia*, and *Ca*. *trachelium* (Fig 1) were cultivated to seedling stage in the greenhouses of the Department of Plant Systematics, University of Bayreuth, and of the Botanical Garden of Ulm, University of Ulm, both in Germany. Subsequently, plants were cultivated outdoors in flowerbeds, where they grew to maturity.

All species selected for this study occur in north hemisphere and, at least in part, their distributions overlap with each other and with that of *Ch*. *rapunculi*. With the exception of *Ca*. *lactiflora*, which has a restricted distribution in the Turkey and Caucasus region [33], all other species are widely spread. *Ca*. *glomerata*, *Ca*. *trachelium* and *Ca*. *rapunculoides* are all common elements in the Eurasian region, *Ca*. *rotundifolia* is distributed throughout the circumpolar region, while *Ca*. *persicifolia* is found predominantly in central Europe [32].

### Color measurements of *Campanula* flowers and bee color hexagon

The spectral reflection properties of the corolla of *Ca*. *glomerata*, *Ca*. *lactiflora*, *Ca*. *persicifolia* (blue and white variety), *Ca*. *rapunculoides*, *Ca*. *rotundifolia*, and *Ca*. *trachelium*, were recorded from 300 to 700 nm (the wavelength perceived by bees; [37]) using a Varian Cary 5 spectrophotometer equipped with a Praying Mantis accessory (Varian, Inc, Palo Alto, California). For each species, measurements were taken from the inner surface of the corolla of three plant individuals (*N* = 1 flower per individual). For each flower, two measurements were taken, one from the basis and the other from the tip of the corolla. Barium sulfate was used as white standard and the disconnected beam as black reference.

The mean reflections of the petals (built first from the measurements of the tip and basis of each flower and then from the three replicates per species) were used to determine the loci of corolla colors in the hexagon color space [38]. We applied the daylight irradiance spectrum D65 as standard and used the spectral sensitivity of honeybee’s photoreceptors [39]. Given that bees do not differ substantially in their visual sensory systems [37], we used the spectral sensitivity functions described for the honeybees as representative approximation for *Chelostoma rapunculi* [39]. The position of the color loci show how bees perceive the corollas through their ultraviolet, blue and green photoreceptors and through further processing of receptor signals in the central nervous system [40]. The color hexagon is separated in six different color sectors representing the different bee color spaces, i.e. UV, UV-blue, blue, blue-green, green, UV-green [41].

For comparison of the bee colors among the different *Campanula* species, the pairwise hexagon distances of color loci among species, as well as the distance of each color locus to its background (green leaves) was calculated [39]. The reflectance function of a typical green leaf was used as background color [41]. We opted to use this reflectance function because *Campanula* species frequently grow together with other plant species, meaning that the background might not represent only the reflectance function of their own leaves. Behavioral experiments with bumblebees trained to visit artificial flowers have demonstrated that color distances of 0.05 hexagon units are poorly discriminated, whereas distances greater than 0.1 are easily discriminated [42, 43].

### Bioassays testing the role of visual cues

#### Pre-testing procedures

To test innate responses of *Ch*. *rapunculi* to visual cues of flowers, we performed a series of behavioral assays using flower-inexperienced bees, which we define here as bees that had no previous contact with flowers. To assure this, trap nests filled with preimaginal *Ch*. *rapunculi* bees were placed at the end of May in an experimental flight cage, where no plants were available. Thus, as bees emerged they did not have the possibility to visit flowers, but rather only black sponge feeders saturated with sugar water (30%, fructose and glucose 1:1). We do not believe that the visual cues perceived by the bees during this time influenced our behavioral assays. Even in the absence of plants, bees flew actively in the flight cage, foraged for sugar water on feeders and mated. The trap nests filled with preimaginal bees were provisioned one year before by females collected at the surroundings of Ulm and Bayreuth, where some of the *Campanula* investigated do not occur naturally. However, during provisioning in the flight cage, individuals of all *Campanula* species were made available for the bees.

The experimental flight cage, located in the botanical garden of Ulm (Germany), consisted of a steel frame (7m x 3.5m x 2.2m large) covered with a fine mesh (stitch density of 1mm x 0.5mm) that was buried into the soil for a deep of 0.5 m. This setup allowed abiotic conditions inside the flight cage (e.g. day length, temperature, air humidity, and light conditions) similar to those found outside in nature.

#### Design of bioassays

To establish whether color cues are involved somehow in the process of host-flower recognition by *Ch*. *rapunculi*, we performed a series of two choice bioassays testing the attractiveness of different colors to flower-inexperienced bees. Behavioral assays were performed first with inflorescences of the white and blue variety of *Ca*. *persicifolia* to reflect a context in which bees are frequently confronted in the nature. By doing this, we expected to test the role of color alone, since size and shape of the blue and white variety of *Ca*. *persicifolia* were similar (Milet-Pinheiro, unp. data). To investigate more accurately the role of color in the process of host recognition, we performed then further bioassays with artificial flowers.

For the two choice bioassay with inflorescences of *Ca*. *persicifolia*, we used transparent solid cylinders (39 cm in height and 9.5 cm in diameter) that allow bees to see, but not to smell the flowers (for a diagram see [44]). The cylinders were made from Makrolon because of its ultraviolet (UV) transparency. Inflorescences of both white and blue variety of *Ca*. *persicifolia* were each covered with a cylinder and offered simultaneously to the bees in the flight cage. The inflorescences of both varieties had six flowers each and were 20 cm in height.

For the bioassays with artificial flowers, we fashioned funnel-shaped flowers (colors: lilac, yellow, and white; dimensions: 4.5 cm in length and 2 cm in diameter at the top). Besides lilac, as a representative color of *Campanula* species, we choose yellow and white because of the representativeness of these colors in the nature [45]. In the flight cage, bees were offered a series of paired combinations of these artificial flowers: 1) white vs. lilac, 2) white vs. yellow, and 3) yellow vs. lilac. For each bioassay, three artificial flowers of each color were presented simultaneously "as an inflorescence" to the bees. The flowers were attached at the base on a thin wooden stick (length: 23 cm; diameter: 2 mm), which was fixed at the ground.

The bioassays with both inflorescences of *Ca*. *persicifolia* and artificial flowers were conducted for 30 min; the initial position of either cylinders or artificial flower groups was inverted after 15 min. Cylinders and groups of artificial flowers were placed 1.5 meter from each other. We always used new artificial flowers in each bioassay to avoid influence of footprints of bees in the decision of other bee individuals. All bees either approaching to (to a distance ≤ 5 cm) or landing on either artificial flowers or cylinders were caught with insect nets and stored in an icebox to avoid pseudo-replications (i.e. counting a response of a single bee more than once). At the end of the experiments, all bees stored in the icebox were released in the flight cage and could participate in subsequent tests. The biotests were performed on sunny days between 1000 h and 1400 h, coinciding with the time of highest foraging activity of bees. About 100 bee individuals (females and males together) were present in the flight cage for the bioassays.

### Sampling of floral scents

To obtain scent samples for the chemical analyses, volatiles were collected from inflorescences of *Ca*. *glomerata*, *Ca*. *lactiflora*, *Ca*. *persicifolia* (blue and white variety), *Ca*. *rapunculoides*, and *Ca*. *rotundifolia* using standard dynamic headspace methods. Floral scents of *Ca*. *trachelium* had been collected and characterized before using the same methods [34]. Fresh inflorescences of three different plants (number of samples and flowers varied among species; Table 1) were enclosed in a polyester oven bag (20 x 30 cm; Toppits). The volatiles were trapped for 4 h in an adsorbent tube, through which air was flown at a rate of 200 mL min^-1^ using a membrane pump (G12/01 EB, Rietschle Thomas, Puchheim, Germany). The adsorbent tubes consisted of ChromatoProbe quartz microvials (GC/MS: length: 15 mm; inner diameter: 2 mm; Varian Inc., Palo Alto, CA, USA), cut at the closed end, and filled with a mixture of 1.5 mg Tenax-TA (mesh 60–80; Supelco, Bellefonte, Pennsylvania, USA) and 1.5 mg Carbotrap B (mesh 20–40, Supelco, Bellefonte, Pennsylvania, USA), which was held in the tubes using glass wool [46].

**Table 1:** Mean absolute and relative amount of volatile compounds in headspace samples collected from inflorescences of six *Campanula* species (one species with two morphs).

Compounds	KRI	GLO	LAC	PER	PEA	RPC	ROT	TRA
**No. of samples; flowers**		5; 235	5; 300	5; 280	7; 389	6; 251	7; 418	6; 379
**No. of compounds**		43	32	29	27	30	41	55
**Amount of scent trapped ng/flower/h**		0.27	4.01	6.01	1.99	1.09	0.72	3.31
**Aliphatics**								
(*Z*)-3-Hexenyl acetate	1006	**12.99**	**12.24**	-	-	-	**55.54**	**16.69**
Hexyl acetate	1013	-	0.13	-	-	-	2.64	-
2-Nonanone*	1094	-	-	-	-	-	-	1.26
Tridecane*	1300	-	-	-	-	-	-	**4.61**
**Aromatics**								
p-Methylanisole*	1025	0.70	-	-	-	-	-	-
Benzyl alcohol*	1039	-	-	-	-	-	2.30	-
Benzeneacetaldehyde*	1050	-	-	3.31	1.78	**61.71**	-	3.40
1-Phenylethanol	1066	-	-	-	-	-	0.46	-
Guaiacol*	1095	0.33	-	-	-	-	-	0.06
2-Phenylethanol*	1121	4.29	0.11	3.90	6.78	**9.87**	**2.68**	**4.60**
Benzyl acetate*	1169	-	-	-	-	-	0.25	-
Methyl saliciyate*	1209	**5.39**	-	-	-	-	**8.75**	0.75
*o*-Anisaldehyde	1253	0.68	-	-	-	-	-	-
2-Phenylethyl acetate*	1263	-	-	-	-	0.31	0.25	0.10
*p*-Anisaldehyde	1266	0.97	-	-	-	0.61	-	-
4-Ethylguaiacol	1287	-	-	-	-	-	0.10	-
**Irregular terpenes**								
4-Oxoisophorone	1151	1.37	0.03	0.38	-	0.47	0.06	-
Geranyl acetone*	1460	-	-	-	-	-	-	0.24
**Monoterpenes**								
α-Pinene*	942	-	-	0.7	3.34	-	-	-
Sabinene*	982	5.09	-	0.99	1.18	-	-	-
ß-Pinene*	985	-	-	0.35	0.72	-	-	-
Myrcene*	995	-	**0.51**	**7.14**	**9.49**	-	0.45	-
δ-3-Carene*	1018	1.06	-	-	-	-	-	0.27
(*Z*)-β-Ocimene*	1040	-	**20.26**	**4.80**	**10.60**	-	-	**4.16**
(*E*)-β-Ocimene*	1052	-	**59.69**	**21.71**	**26.67**	-	**7.33**	**38.57**
(*E*)-Linalol oxide (furanoid) *	1094	-	-	1.00	1.53	**9.67**	-	0.05
Terpinolene*	1096	0.46	-	0.19	0.41	-	-	0.07
Linalool*	1103	-	-	3.76	-	-	-	1.95
Allo-ocimene	1132	-	**0.64**	2.13	4.64	-	-	-
*p*-Cymene*	1133	-	-	-	-	-	-	0.43
(*E*)-Epoxy-ocimene	1144	-	0.08	0.45	0.25	-	-	0.28
Neo-allo-ocimene	1145	-	0.01	0.42	0.16	-	-	-
Dill ether	1162	-	0.15	-	-	-	-	-
*p*-Mentha-1,5-dien-8-ol	1176	-	0.09	-	-	-	-	-
(*Z*)-Linalool oxide (pyranoid) *	1181	-	-	-	-	**4.21**	-	-
α-Terpineol*	1203	0.63	-	0.87	2.44	-	0.20	0.03
Verbenone*	1227	-	-	-	-	-	-	0.05
(*E*)-Ocimenone	1237	-	0.06	-	-	-	-	-
Lavandulyl acetate	1289	0.17	-	-	-	-	0.41	-
**N-compounds**								
Benzeneacetonitrile*	1147	1.69	-	0.92	0.35	0.72	-	-
Indole*	1304	-	-	-	-	0.67	0.11	-
1-Nitro-2-phenylethane*	1309	0.71	-	-	-	0.18	-	-
*o*-Aminoacetophenone	1313	-	0.37	-	-	0.23	-	-
**Sesquiterpenes**								
α-Cubebene	1365	1.59	-	-	-	0.40	0.07	0.23
α-Longipinene	1379	-	-	0.54	0.43	-	-	0.12
α-Ylangene	1389	2.04	-	**14.03**	**8.47**	**2.00**	1.43	0.68
α-Copaene*	1395	**6.47**	-	-	-	1.17	0.69	0.34
Longifolene	1402	-	-	-	-	-	-	0.08
ß-Bourbonene	1407	-	-	1.19	0.29	1.67	0.94	-
ß-Elemene	1407	-	-	-	-	-	-	1.99
ß-Cedrene	1441	0.52	-	-	-	-	0.22	0.59
(*E*)-ß-Caryophyllene*	1445	5.16	-	**14.82**	**9.50**	-	**7.08**	0.64
(*E*)-α-Bergamotene*	1454	-	-	-	-	-	-	2.08
(*E*)-ß-Farnesene*	1460	2.05	0.30	-	-	0.50	0.08	1.79
α-Caryophyllene*	1480	0.45	0.18	-	-	-	1.51	-
Prezizaene	1484	-	-	-	1.07	-	0.03	1.58
Amorpha-4,11-diene	1485	-	-	-	-	-	-	0.80
Allo-aromadendrene*	1488	0.64	-	-	-	0.43	0.09	-
ß-Cubenene	1493	0.12	-	-	-	0.13	-	-
Ar-Curcumene	1494	-	-	-	-	-	Tr	0.95
γ-Muurolene	1496	0.24	-	-	-	0.38	0.09	-
(*Z*, *E*)-α-Farnesene*	1496	0.27	-	-	-	0.15	-	0.42
Germacrene D*	1506	**12.36**	-	0.99	0.41	-	2.55	0.70
(*E*, *E*)-α-Farnesene*	1510	-	0.06	1.30	0.28	-	-	-
ß-Selinene*	1517	-	-	-	-	-	-	0.78
α-Muurolene*	1527	-	-	-	-	-	-	1.35
α-Selinene	1529	-	-	-	-	-	-	0.50
(*Z*)-γ-Bisabolene	1535	-	-	-	-	-	-	0.69
ß-Sesquiphellandrene	1542	-	-	-	-	-	-	0.69
δ-Cadinene*	1542	1.36	-	-	-	-	0.26	0.42
α-Calacorene	1567	-	-	-	-	-	-	0.12
19 Unknown sesquiterpenes		13.12	-	13.63	8.79	3.53	3.17	1.42
**Spiroacetals**								
1,6-Dioxaspiro[4.5]decane*	1057	-	-	0.02	-	-	-	0.01
(*E*)-7-Methyl-1,6-dioxaspiro[4.5]decane (*E*-Conophthorin) *	1065	**5.84**	-	0.09	0.21	0.01	0.03	0.41
(*Z*)-7-Methyl-1,6-dioxaspiro[4.5]decane (*Z*-Conophthorin) *	1140	0.41	-	-	-	-	Tr	-
(*E*)-2-Methyl-1,7-dioxaspiro[5.5]undecane*	1152	-	-	-	-	-	-	0.14
(*E*)-7-Ethyl-1,6-dioxaspiro[4.5]decane*	1156	0.31	-			0.16	-	0.93
(*Z*)-7-Ethyl-1,6-dioxaspiro[4.5]decane*	1231	-	-	-	-	-	-	0.08
**Unknowns**								
22 compounds		10.48	5.09	0.34	0.21	0.81	0.23	1.86

* Identification based on authentic standards.

Volatiles are listed according to chemical class and within class according to Kovats Retention Indices (KRI). Tr—compounds found in relative amount < 0.01%. Marked in bold are the five most abundant compounds of each species. Abbreviations of species names—*Campanula glomerata* (GLO), *Ca*. *lactiflora* (LAC), *Ca*. *persicifolia* (PER), *Ca*. *persicifolia alba* (PEA), *Ca*. *rapunculoides* (RPC), *Ca*. *rotundifolia* (ROT), and *Ca*. *trachelium* (TRA). The original data on the flower scent of *Ca*. *trachelium* are published in Milet-Pinheiro *et al*. [34].

To control for non-floral (vegetative) volatiles and contaminants in the floral scent samples, headspace samples of non-flowering plants (*N* = 3 for each species) and blank controls (empty oven bags; *N* = 3 for each species) were also collected following the same methods as described above. All headspace samples were stored in screw cap vials at -20°C until chemical analyses.

### Chemical analyses of floral scent samples

The headspace samples were analyzed on a Varian Saturn 2000 mass spectrometer coupled to a Varian 3800 gas chromatograph (GC/MS) equipped with a 1079 injector (Varian Inc., Palo Alto, CA, USA), which had been fitted with the ChromatoProbe kit (see [46]). A quartz microvial was loaded into the probe, which was then inserted into the modified GC injector. The injector split vent was opened and the injector heated to 40°C to flush any air from the system. After 2 min, the split vent was closed, and the injector heated to 200°C/min at a rate of 200°C min^-1^. This temperature was then held at 200°C for 4.2 min, after which the split vent was opened and the injector cooled down. Separation of compounds was achieved with a fused silica column ZB-5 (5% phenyl polysiloxane; 60 m long, inner diameter 0.25 mm, film thickness 0.25 μm, Phenomenex). Electronic flow control was used to maintain a constant helium carrier gas flow of 1.0 mL min^-1^. The GC oven temperature was held for 7 min at 40°C, then increased by 6°C per min to 250°C and held for 1 min. The MS interface worked at 260°C and the ion trap at 175°C. Mass spectra were taken at 70 eV (in EI mode) with a scanning speed of 1 scan sec^-1^ from m/z 30 to 350. The GC/MS data were processed using the Saturn Software package 5.2.1.

Identification of compounds was carried out using the NIST 08, Wiley 7, and Adams [47] mass spectral data bases, or the data base provided in MassFinder 3, and confirmed by comparison of retention times with published data [47]. Structure assignments of individual components were confirmed by comparison of mass spectra and GC retention times with those of commercially available standards.

The composition of volatiles collected from flowering plants was compared with that from non-flowering plants (vegetative parts). Floral scent volatiles were those detected either in higher amounts or exclusively in floral scent headspace samples. Volatiles detected in the empty bags were considered ambient contaminants and were omitted from the experimental samples.

### Statistical analyses

To test for differences in bee responses between the paired treatments in each behavioral assay, we performed two-tailed exact binomial tests. Binomial tests were calculated using the spreadsheet provided by http://www.biostathandbook.com/exactgof.html (accessed 02 July 2013; see also [48]). Responses of male and female bees were pooled, as individuals of both sexes have been shown to respond similarly to artificial flowers and to visual cues of host flowers (Fisher's Exact tests: 0.21 < *P* < 0.71). The similar behavior of male and female of *Ch*. *rapunculi* to floral cues of host flowers had already been evidenced in a previous work [20].

Flower scent profiles of the investigated species were compared using both qualitative (presence/absence of compounds) and semi-quantitative (relative amount of compounds with respect to total peak area) approaches. For qualitative and semi-quantitative comparisons, we calculated the Sørensen and Bray-curtis similarity indices, respectively. These indices determine pairwise similarities among the individual samples. The relative ratios of compounds were transformed to their square root for the semi-quantitative analysis. After that, and based on the obtained similarity matrices (individual based matrices), we performed analyses of similarities (ANOSIM, 10,000 permutations) to test for differences in scent bouquet among species. ANOSIM is a commonly used multivariate procedure roughly analogous to ANOVA/MANOVA that operates directly on a (dis)similarity matrix. It yields a test statistic R that is a relative measure of separation among a priori defined groups. It is based on differences of mean ranks among and within groups. A R value of ‘0’ indicates completely random grouping, whereas a value of ‘1’indicates that samples within groups are more similar to each other than to any sample from a different group [49]. Non-metric multidimensional scaling (NMDS), based on the similarity matrices generated, were used to display graphically the qualitative and semi-quantitative differences in scent profile among species. Stress values indicate how well the two-dimensional plot represents relationships among samples in multidimensional space. Stress values below 0.15 indicate a good fit [49]. The software Primer 6.1.6 was used to calculate the similarity indices of Sørensen and Bray-Curtis and to perform the ANOSIM and NMDS analyses [49].

## Results

### Comparisons of floral color among *Campanula* species and artificial flowers

**Fig 2:**
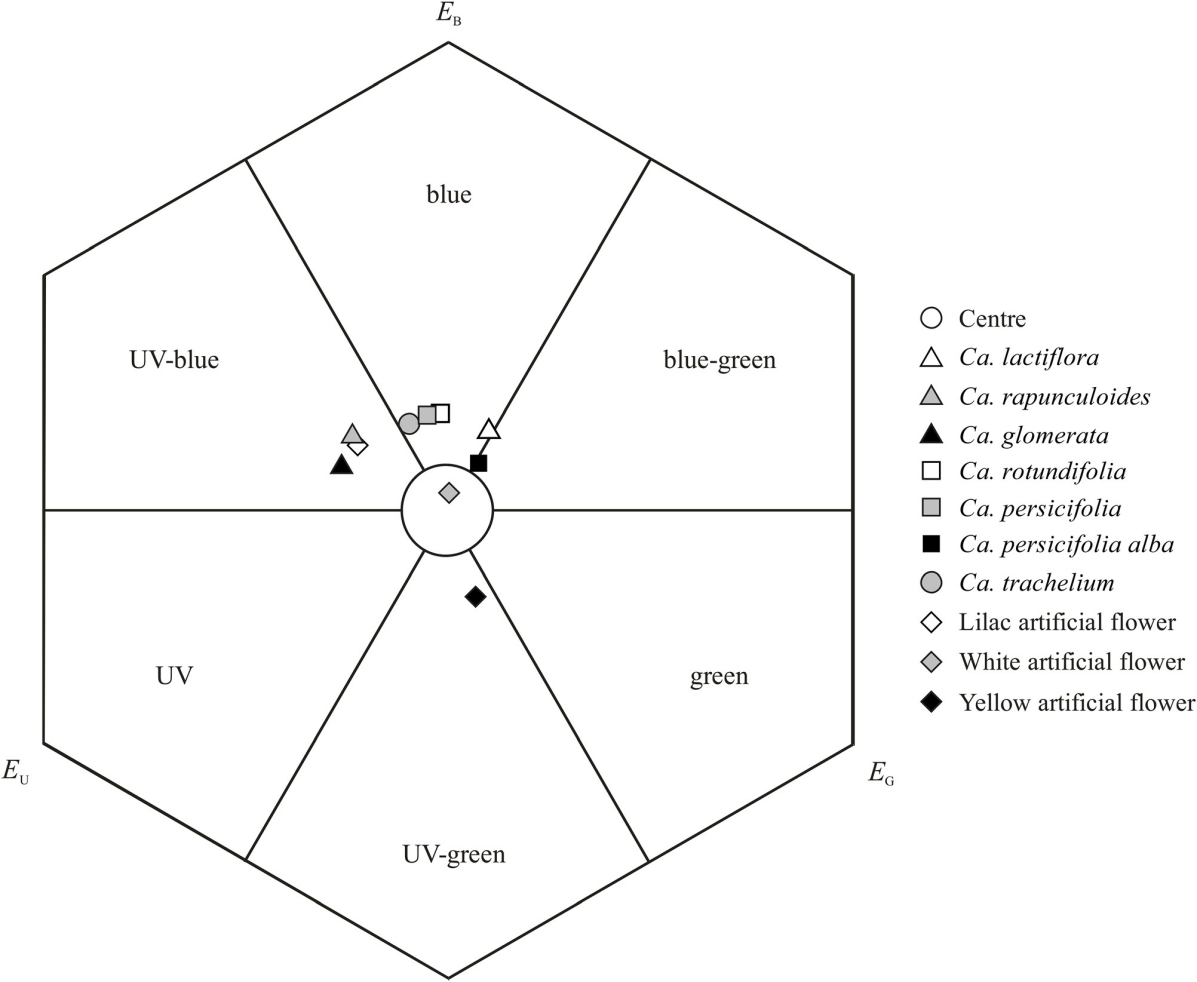
Corolla color loci of *Campanula* in the bee color hexagon. Corolla color loci of six *Campanula* species (one species with two color morphs) and artificial flowers plotted in a hexagon color space. *E*_U_, *E*_B_, *E*_G_: excitation of the UV, blue, and green receptor, respectively. The pairwise interspecific distances in color loci and the distances to the centre of the single species can be found in Supporting Information (S1 Table).

The comparison of corolla reflectance in *Campanula* species showed that the color loci of the investigated species form a gradient across the UV-blue- and blue-green color space in the bee-color hexagon (Fig 2). Taking into account the pairwise interspecific distances in color loci of *Campanula* flowers (S1 Table), three groups can be observed: 1) the UV-blue group, including *Ca*. *glomerata* and *Ca*. *rapunculoides*; 2) the blue group, including *Ca*. *persicifolia*, *Ca*. *rotundifolia*, and *Ca*. *trachelium*; and 3) the blue/blue-green intersection group, including *Ca*. *lactiflora* and *Ca*. *persicifolia alba*. Species within groups reflect color very similarly (i.e. the Euclidean distances in the color hexagon are < 0.1 units), while differing from all other *Campanula* species (i.e. Euclidean distances in the color hexagon are > 0.1 units). The corolla color of all species is easily detectable by bees against a green standard background (hexagon units > 0.1), when assuming that *Chelostoma rapunculi* behave the same as bumble bees (see Materials and Methods).

The color of lilac artificial flowers used in the bioassays resembles that of the flowers of *Ca*. *glomerata* and *Ca*. *rapunculoides* (hexagon units < 0.1). The color loci of the yellow and the white artificial flowers were in the UV-green and uncolored section of the hexagon, respectively. While the white artificial flowers bore some color similarity to flowers of *Ca*. *persicifolia alba* (hexagon units < 0.1), the yellow artificial flowers bore no color similarity to any of the *Campanula* flowers (> 0.1). Bees can discriminate colors of all artificial flowers among each other (S1 Table).

### Behavioral assays

**Fig 3:**
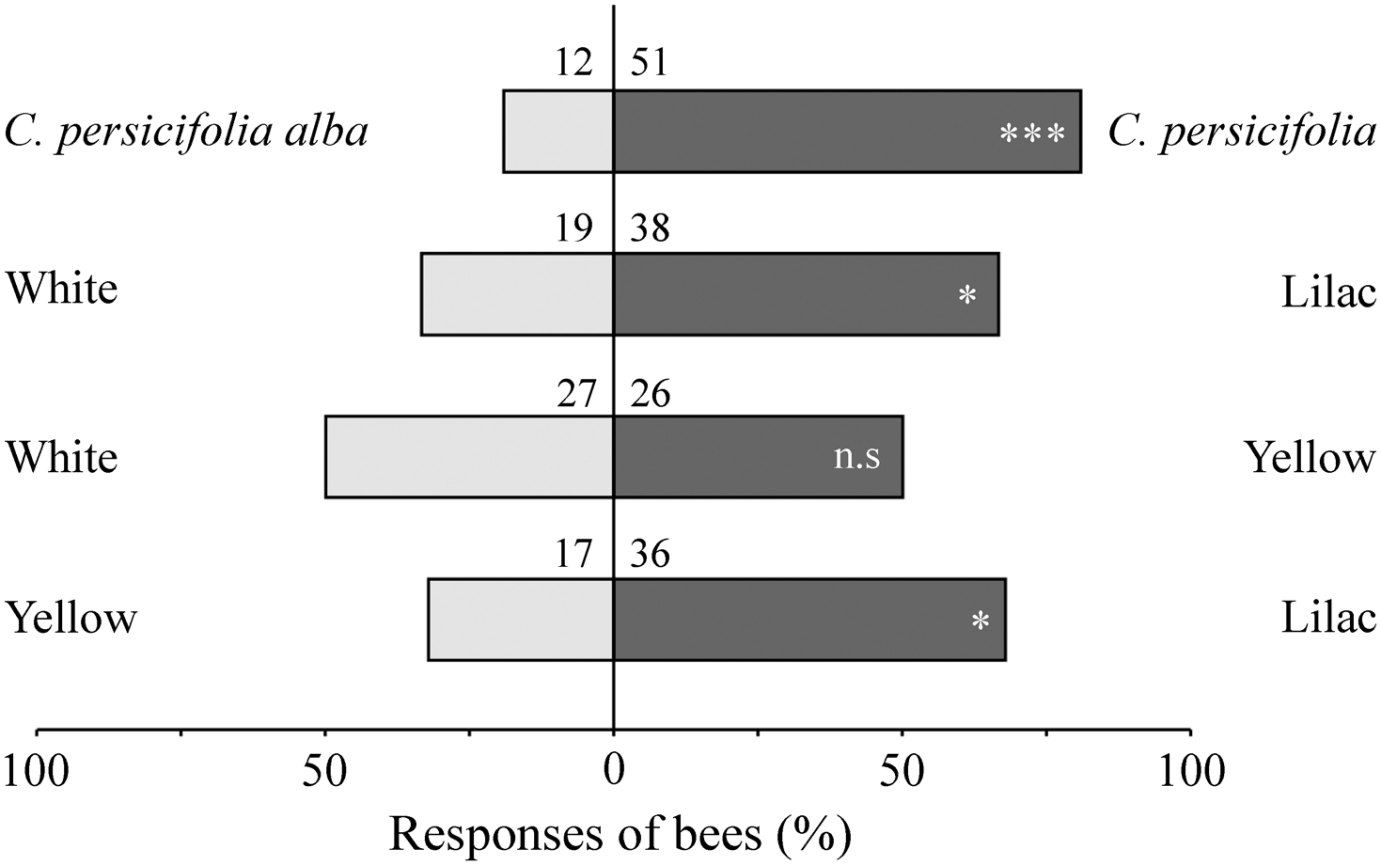
Behavioral assays testing attractiveness of inflorescences and artificial flowers to *Chelostoma rapunculi* bees. Responses of flower-inexperienced bees of *Chelostoma rapunculi* (males and females together) to visual cues of either inflorescences of *Campanula persicifolia* or artificial flowers in behavioral assays performed in an experimental flight cage. Numbers above bars indicate the absolute number of responding bees. Difference in responses for each assay was assessed by an exact binominal test, *P* < 0.05 (*), *P* < 0.001 (***), not significant (n.s).

For the bioassays with inflorescences, flower-inexperienced bees showed a clear preference for the visual cues of *Ca*. *persicifolia* over those of *Ca*. *persicifolia alba* (Fig 3).

The innate preference of *Ch*. *rapunculi* for a color characteristic for *Campanula* was confirmed in the bioassays using artificial flowers. Lilac artificial flowers were more attractive than both yellow and white flowers, whereas no preference was detected when yellow and white flowers were tested against each other (Fig 3).

### Comparison of floral scents among *Campanula* species

The total amount of scent trapped per flower and per hour ranged from 0.7 ng in *Ca*. *rotundifolia* to 6 ng in *Ca*. *persicifolia* (Table 1). We detected 118 compounds in the scent bouquet of the different *Campanula* species, from which 77 could be identified (Table 1; S2 Table). Altogether, representatives of seven compound classes were recorded in the different *Campanula* species: sesquiterpenes (47 compounds), monoterpenes (21), aromatics (12), spiroacetals (6), aliphatics (4), N-compounds (4), and irregular terpenes (2); the class of 22 compounds could not be determined. *Campanula glomerata* and *Ca*. *rotundifolia* had representatives from all compound classes, while *Ca*. *persicifolia*, *Ca*. *persicifolia alba*, *Ca*. *rapunculoides*, and *Ca*. *trachelium* from six. The number of floral volatile compounds produced by each species varied considerably, ranging from 27 in *Ca*. *persicifolia alba* to 55 in *Ca*. *trachelium*. There was only one compound, namely 2-phenylethanol, which was recorded in all analyzed species. Additional compounds frequently recorded were (*E*)-conophthorin, 4-oxoisophorone, and α-ylangene (in five species each). The scent bouquet of the *Campanula* species was generally dominated by a few compounds emitted in large amounts and several compounds emitted in low amounts. The relative contribution to the total flower scent discharge of the five most abundant compounds of each species ranged from 42.4% in *Ca*. *glomerata* to 93.3% in *Ca*. *lactiflora* (Table 1).

Spiroacetals (at least two per species) were recorded in all species, except for *Ca*. *lactiflora*. The most common spiroacetal was (*E*)-conophthorin (recorded in five species) followed by (*E*)-7-Ethyl-1,6-dioxaspiro[4.5]decane (3 spp.), and 1,6-Dioxaspiro[4.5]decane and (*Z*)-conophthorin (2 spp. each). (*E*)-7-Ethyl-1,6-dioxaspiro[4.5]decane and (*E*)-2-Methyl-1,7-dioxaspiro[5.5]undecane were recorded in only one species each. Spiroacetals were minor compounds in all species, normally responding to relative amounts lesser than 1%, with the exception of (*E*)-conophthorin in *Ca*. *glomerata* that responded to about 6% of the total scent discharge. None of the spiroacetals was recorded in all samples of the species in which they were reported (S2 Table).

**Fig 4:**
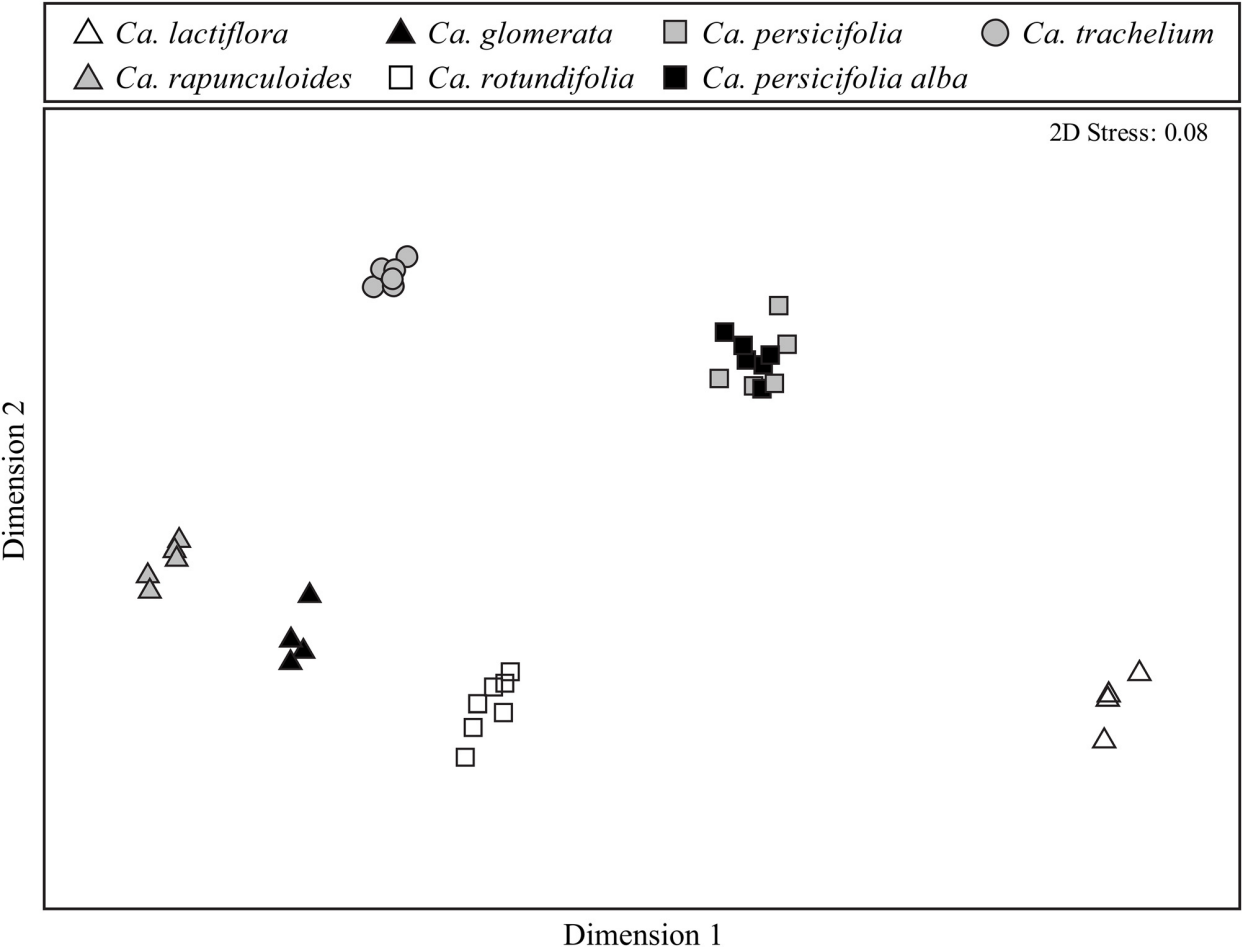
Nonmetric multidimensional scaling (NMDS) of floral scent composition in *Campanula*. Comparison of floral scent bouquets among six *Campanula* species (one species with two color morphs) based on qualitative Bray—Curtis similarities plotted in a non-metric multidimensional scaling (NMDS). The NMDS plot based on semi-quantitative similarities is not shown since samples clustered similarly (see text).

Multivariate analyses evidenced significant differences in both qualitative (presence/absence of compounds; ANOSIM global *R* = 0.982, *P* < 0.01) and semi-quantitative (relative amount of compounds; ANOSIM global *R* = 0.985, *P* < 0.01) composition of floral scents among species. Composition of floral scents differed significantly for all pairs of species tested (*R* = 1 for all pairs and *P* < 0.05 for both qualitative and semi-quantitative comparisons), meaning that each species produces its own scent profile (Fig 4). Qualitative (*R* = 0.68, P < 0.01) and semi-quantitative differences (*R* = 0.76, *P* < 0.01) in scent composition were evident even between the blue and white variation of *Ca*. *persicifolia*. With the exception of *Ca*. *persicifolia*, floral scent samples of the *Campanula* species used by *Ch*. *rapunculi* as pollen source were plotted more close to each other when compared to the samples of the non-host plant *Ca*. *lactiflora* (Fig 4).

## Discussion

In the present study, we hypothesized that *Campanula* species share visual and olfactory floral cues that could be used as host-recognition cues by oligolectic bees of *Ch*. *rapunculi*. In corroboration to our hypothesis, we found that the flowers of most *Campanula* species were grouped into the UV-blue and blue bee-color space and that these colors are more attractive than others (e.g. UV-green and blue-green) to the *Campanula* pollen-specialist bee *Ch*. *rapunculi*. In terms of olfactory cues, we found that floral scent bouquets are species-specific and that highly specific volatiles, i.e. spiroacetals, which were shown to be the key compounds in *Ca*. *trachelium* for host recognition of *C*. *rapunculi* [34], are emitted by all species investigated with the exception of *Ca*. *lactiflora*. Interestingly, among the *Campanula* species investigated, this is the only one that is not used by *Ch*. *rapunculi* females as pollen source.

### Floral color in C*ampanula* and its significance for host location by *Chelostoma rapunculi* bees

The color analyses performed in this study evidenced that the corolla floral reflectance of the *Campanula* species form a gradient across the UV-blue and blue-green bee-color space. These results are in congruence with those in the floral reflectance database (FReD; [45]), where seven of the ten further *Campanula* species investigated were shown to reflect the light in the UV-blue, two in the blue, and one in the blue-green bee-color space. This clearly shows that the UV-blue and blue are the most representative bee-colors in the genus *Campanula*. Color is crucial in flower location by bees as a whole [2, 12] and in the perspective of the oligolectic pollinator *Ch*. *rapunculi* it seems to have a strong significance for host location. The set of bioassays showed that flower-inexperienced bees of *Ch*. *rapunculi* have an innate preference exactly for the two bee-color categories which are most representative for flowers of their *Campanula* host plants. *Chelostoma rapunculi* is a strict oligolectic species that does not provide nests in the absence of *Campanula* host flowers [20, 34]. Therefore, host location is crucial for a successful brood provision and our findings indicate that color has a strong significance in this sense, i.e. the innate color preference of *Ch*. *rapunculi* would tune its foraging activity to UV-blue/blue colors, increasing the chance of locating *Campanula* host flowers. While our results clearly indicate that color is important for host location by *Ch*. *rapunculi*, its significance for host recognition is questionable. The UV-blue and blue-bee colors of *Campanula* flowers is shared by several other plants belonging to different genera and families (see the floral reflectance database, [45]), suggesting that color alone would not be specific enough to allow unambiguously host recognition (see also [20, 26, 50]). Thus, further cues other than color might be necessary to allow recognition of *Campanula* host flowers by *Ch*. *rapunculi*.

### Floral scent in *Campanula* and its significance for host recognition by *Chelostoma rapunculi* bees

In recent investigations, we showed that newly-emerged *Ch*. *rapunculi* bees differentiate *Ca*. *trachelium* from non-host species of other plant groups using floral scents and that unusual floral scent compounds, i.e. spiroacetals, are used by these bees for host recognition [20, 34]. Since *Ch*. *rapunculi* bees visit several *Campanula* species to collect pollen, we hypothesized that the presence of spiroacetals would be a common trait typifying *Campanula* host plants. In corroboration with this hypothesis, we found spiroacetals as constituents of the scent bouquet of five among the six species of *Campanula* investigated. Not surprisingly, the only species that does not emit spiroacetals, i.e. *Ca*. *lactiflora*, has never been reported as pollen source of *Ch*. *rapunculi*. In support of this finding, *Ch*. *rapunculi* bees did not visit flowers of *Ca*. *lactiflora* in our flight cage, even in the absence of host plants (Milet-Pinheiro, unp. data). Spiroacetals are relatively widespread volatiles in nature, occurring, for example, in mammals [51], insects [52], and in the bark of many conifer trees [53–55]. In contrast, they are rarely encountered in floral scents [24], and thus might give a distinct, unique floral scent identity to the *Campanula* species that are visited by *Ch*. *rapunculi*.

In the present study, we recorded six spiroacetals, of which four (i.e. 1,6-Dioxaspiro [4.5] decane, *E*-2-Methyl-1,7-dioxaspiro[5.5]undecane, *E*-7-ethyl-1,6-dioxaspiro[4.5]decane, *Z*-7-Ethyl-1,6-dioxaspiro[4.5]decane), have never been reported as constituents of floral scents in plants other than *Campanula* [34]. In contrast, the (*E*)- and (*Z*)-conophthorin isomers are reported in the floral scents of representatives of 13 and 5 plant families, respectively [24]. Curiously, (*E*)-conophthorin was the unique of these compounds emitted by all host plants of *Ch*. *rapunculi*, meaning that this would be the most host-typifying spiroacetal. In this scenario, one might argue that this compound would not be host-specific enough for allowing unambiguous recognition by *Ch*. *rapunculi*. A more comprehensive screening, however, indicates that (*E*)-conophthorin is rather unusual in the context in which *Ch*. *rapunculi* forages; the plants that emit this compound as floral scent constituent either do not occur syntopically (most are tropical representatives of Lecythidaceae, Passifloraceae, Orchidaceae, Moraceae, and Solanaceae) or do not bloom simultaneously with host plants of *Ch*. *rapunculi*. This context-dependent specificity of rather common compounds has also been found in other associations involving oligolectic bees, such as *Andrena vaga* (Andrenidae), *Protodiscelis palpalis* (Colletidae), and *Peponapis pruinosa* (Apidae). In these cases, the key compounds involved in the attraction of the bees, respectively, 1,4-dimethoxybenzene [56], p-methylanisole [57], and 1,2,4-trimethoxybenzene [58] are reported in about 15, 20, and 5 families, respectively [24]. Together, the findings of these studies suggest that compounds, which are not necessarily unique as floral scent constituents in general, may be very unusual in certain context and indeed are involved in host-recognition by oligolectic bees.

The possibility that spiroacetals other than conophthorin are also involved in the host-recognition of *Campanula* flowers by host-naive *Ch*. *rapunculi* bees cannot be ruled out, since the attractiveness of these compounds was tested as a mixture [34]. Furthermore, although recorded in all species, (*E*)-conophthorin was absent in some samples. Similarly, the other spiroacetals were never recorded in all scent samples of a given species. This suggests that spiroacetals may be either not emitted by all plant individuals or emitted in amounts below the detection limit of the GC-MS. We believe that the second alternative is more likely, since spiroacetals are normally minor constituents of the astonishing weak floral scent bouquet of *Campanula* species. Additionally, electroantennographic analyses performed with *Ch*. *rapunculi* indicate that these bees can detect spiroacetals in concentrations as low as 1:10000000, a concentration that is below the detection limit of the GC-MS used for the scent analyses (Milet-Pinheiro *et al*. in prep.). Thus, it is well possible that *Ch*. *rapunculi* uses the other spiroacetals or a combination thereof to recognize host plants. The high specificity of all these compounds, mainly if they act in combination, would make host plants of *Ch*. *rapunculi* even more unambiguous. Behavioral assays testing the role of spiroacetals individually and in mixtures are necessary to shed more light on this complex and challenging puzzle of host recognition by *Ch*. *rapunculi*.

While the argumentation above may help to explain the absence of spiroacetals in some samples of the *Campanula* species that host *Ch*. *rapunculi*, it also brings some hesitation for their absence in the non-host *Ca*. *lactiflora*. However, different to the host plants of *Ch*. *rapunculi*, spiroacetals were not detected in any of the samples of *Ca*. *lactiflora* (even in trace amounts), in spite of the great number of individuals and flowers used, suggesting that they are indeed not produced by flowers of *Ca*. *lactiflora*. Additional support of the absence of spiroacetals in *Ca*. *lactiflora* is provided by the phylogeny. This species belongs to a clade that does not include host plants [59, 60]. Future studies should test for the presence of spiroacetals in other non-host species.

Studies investigating the role of floral scent in host recognition by oligolectic bees are very scarce. Nevertheless, the general tendency emerging so far suggests that oligolectic bees rely initially on a single or few compounds to recognize host flowers [26, 34, 50, 56, 57]. Oligolectic bees generally collect pollen from different species within a clade [61], which may vary considerably in floral scent composition [19, 21]. In the case of *Campanula*, the chemical analyses showed that both qualitative (presence/absence) and semi-quantitative (relative amount) scent composition of *Campanula* are species-specific, i.e. each species has its own scent profile. In consequence, single or few compounds that are shared by host plants might represent a much more predictable and reliable cue for host recognition by oligolectic bees in their first foraging trips than complex scent bouquet.

Interestingly, increasing evidences suggest that the innate chemical search-image of oligolectic bees changes to a more complex mixture of volatiles after bees acquire foraging experience on host flowers [11], indicating that bees might benefit of adding further compounds to their innate search-image. Pollen and nectar are known to be scented and in some cases its emitted volatiles can be distinguished from those of other flower parts [26, 62]. In the case of *Ca*. *trachelium*, scent bouquets of floral parts containing nectar and pollen are dominated by linalool and (*E*)-ß-ocimene, which are only attractive after foraging experience [34]. If *Ch*. *rapunculi* bees learn to associate these compounds (or other compounds) with pollen and/or nectar availability, adding them to their chemical search-image might facilitate selection of host flowers with plenty of resources, thereby increasing foraging efficiency (see also [29]). In the perspective of the plants, associative learning might assure flower constancy by pollinators with the benefit of reducing pollen loss while bees visit flowers of other species. Consequently, the species-specific nature of floral scents might be of great significance for plant reproductive isolation mainly among syntopic species, as is the case for several *Campanula* species. Further experimental studies are still necessary to establish to what extent changes in innate chemical search-image are mediated by associative learning in this and other oligolectic bees and to understand how this phenomenon affects reproductive isolation of plants.

### Integration of visual and olfactory cues

As suggested above, visual and olfactory cues seem to play different, but synergistic roles, in the process of host recognition by *Ch*. *rapunculi*. In a previous study, we showed that decoupled visual and olfactory cues are attractive to *Ch*. *rapunculi*, but that a combination of these two sensory modalities is much more attractive than is either cue alone [20]. Furthermore, decoupled visual cues were found to be more attractive for bees than olfactory cues when these stimuli were tested against each other. This suggests that foraging of *Ch*. *rapunculi* bees is based first on visual cues, after which floral scents might come into play, and evidences the importance of multisensory integration for recognition of host flowers. Thus, as supported by our experiment, color might be interpreted as a foraging filter, which leads bees to preferentially approach UV-blue/blue bee-colored flowers, while floral scents provide more specific information, such as host identity and resource availability (see also [44]).

Multisensory integration in bees is believed to impact positively on foraging efficiency, by improving aspects such as accuracy, decision speed, and learning speed [13]. Besides in *Ch*. *rapunculi*, the impact of multisensory integration for host-flower recognition, as compared to single sensory modalities, has been tested in a few oligolectic species, namely *Hoplitis adunca* [19] and *Macropis fulvipes* [63]. The innate relative reliance on visual as compared to olfactory cues has been found to vary considerably between species. In *H*. *adunca*, for example, female bees respond to visual, but not to olfactory cues of host flowers. In contrast, *M*. *fulvipes* females respond to olfactory, but not to visual cues. In spite of the relative reliance on decoupled cues, the combination of visual and olfactory cues resulted always in an increased attractiveness to bees as compared to either cues alone in both species and this was true for host-naive and host-experienced bees. Overall, these studies indicate that the impact of decoupled visual and olfactory cues varies from one species to the other, but not the impact of these two cues together. This means that oligolectic bees might benefit of integrating different sensory modalities when foraging on the field.

## Conclusions and Perspectives

Our results, together with those of recent studies investigating the basis of host recognition by *Ch*. *rapunculi*, suggest that this bee has neurological adaptations (vision and olfaction) that innately drive its foraging flights toward host flowers. Furthermore, the strong innate preference displayed by *Ch*. *rapunculi* to both olfactory and visual cues of *Campanula* provide strong evidence that host-plant preference is genetically based (see also [64]); while olfactory cues may be learned during the larval stage [65], visual cues (e.g. color) can only be learned during flower visits. Collectively, these findings strengthen the hypothesis that host-plant choice is genetically constrained in bees and that specific neurophysiological adaptations to host flowers may preclude oligolectic bees of collecting pollen in alternative hosts, thereby restricting diet breadth [8]. In the case of *Ch*. *rapunculi*, the strong innate preference for UV-blue/blue bee-color, but mainly for host-specific spiroacetal volatiles, might be preventing this bee to forage for pollen on plants other than *Campanula*. This is particularly critical for this bee species because its larvae do not develop (or experience high mortality) when reared on pollen of non-host plants [7].

This study represents a significant progress in the current understanding of the floral scent chemistry in the genus *Campanula*, as well as in its ecological significance, and opens interesting perspectives. In Central Europe, the spectrum of bees that visit flowers of *Campanula* is very diverse and includes both pollen-generalists, such as Western honeybee and several bumblebee species, and pollen-specialist species, among others *Andrena curvungula* and *A*. *rufizona* (Andrenidae), *Chelostoma rapunculi*, *Ch*. *campanularum*, *Ch*. *distinctum* (Megachilidae), *Dufourea dentiventris*, *D*. *inermis* (Halictidae), and *Melitta haemorrhoidalis* (Melittidae) [30, 59, 66]. In this scenario, it would be very interesting to investigate in a comparative approach how floral scents of *Campanula* are perceived by polylectic and oligolectic pollinators. Especially interesting would be a comparison in the sensitivity of polylectic and oligolectic species to both ubiquitous (e.g. 2-phenyl ethanol, ocimene) and unusual compounds (spiroacetals) using electroantennographic analyses and behavioral assays. Given the highly specific nature of spiroacetals in *Campanula* flowers, and their significance in host recognition by *Ch*. *rapunculi*, we believe that these volatiles might also be involved in host recognition by other *Campanula* oligoleges. In contrast, polylectic bees, which are less restrictive in pollen diet, might use ubiquitous compounds that represent a broader spectrum of flowering plants. Such a comparative study would give us a stronger grasp not only on the evolution of olfactory receptors in specialized and generalized pollinators but also of floral scents in the genus *Campanula*.

## Supporting Information

S1 Table Euclidean distances among flower color loci of *Campanula* species and artificial flowers. (DOC)

S2 Table Mean absolute and relative amount (%) of volatile compounds in all headspace samples collected from inflorescences of six *Campanula* species. (XLS)
