# Supplementary material for: Visual and Olfactory Floral Cues of Campanula (Campanulaceae) and Their Significance for Host Recognition by an Oligolectic Bee Pollinator
Source: PLoS One. 2015 Jun 10;10(6):e0128577. doi: 10.1371/journal.pone.0128577 (PMC4465695; doi:10.1371/journal.pone.0128577)
Supplement: S1 Table — (DOC) [file pone.0128577.s001.doc]

# Supporting information

**S1 Table.** **Euclidean distances (in hexagon units) among flower color loci of *Campanula* species (values ≤ 0.1 units are in bold) and artificial flowers.** Distances to the hexagon centre are also given. Abbreviations of species names - *Campanula glomerata* (GLO), *Ca. lactiflora* (LAC), *Ca. persicifolia* (PER), *Ca. persicifolia alba* (PEA), *Ca. rapunculoides* (RPC), *Ca. rotundifolia* (ROT), and *Ca. trachelium* (TRA). Abbreviations of artificial flowers - lilac (LIL), yellow (YEL), white (WHI). Hexagon Centre (CEN).

|  | GLO | LAC | PER | PEA | RPC | ROT | TRA | LIL | YEL | WHI | CEN |
| --- | --- | --- | --- | --- | --- | --- | --- | --- | --- | --- | --- |
| GLO |  |  |  |  |  |  |  |  |  |  | 0.25 |
| LAC | 0.33 |  |  |  |  |  |  |  |  |  | 0.20 |
| PER | 0.21 | 0.14 |  |  |  |  |  |  |  |  | 0.21 |
| PEA | 0.29 | **0.07** | 0.15 |  |  |  |  |  |  |  | 0.12 |
| RPC | **0.07** | 0.30 | 0.17 | 0.28 |  |  |  |  |  |  | 0.26 |
| ROT | 0.23 | 0.12 | **0.02** | 0.14 | 0.19 |  |  |  |  |  | 0.21 |
| TRA | 0.17 | 0.17 | **0.04** | 0.17 | 0.12 | **0.06** |  |  |  |  | 0.20 |
| LIL | **0.06** | 0.28 | 0.16 | 0.26 | **0.03** | 0.18 | 0.12 |  |  |  | 0.23 |
| YEL | 0.40 | 0.36 | 0.40 | 0.28 | 0.44 | 0.40 | 0.40 | 0.41 |  |  | 0.19 |
| WHI | 0.24 | 0.16 | 0.17 | **0.08** | 0.24 | 0.16 | 0.17 | 0.22 | 0.24 |  | **0.04** |
